# Hesitant or Not Hesitant? A Systematic Review on Global COVID-19 Vaccine Acceptance in Different Populations

**DOI:** 10.3390/vaccines9080873

**Published:** 2021-08-06

**Authors:** Maria Giulia Salomoni, Zeno Di Valerio, Elisa Gabrielli, Marco Montalti, Dario Tedesco, Federica Guaraldi, Davide Gori

**Affiliations:** 1 Unit of Hygiene, Department of Biomedical and Neuromotor Sciences, Public Health and Medical Statistics, University of Bologna, 40126 Bologna, Italy; mariagiulia.salomoni@studio.unibo.it (M.G.S.); zeno.divalerio@studio.unibo.it (Z.D.V.); elisa.gabrielli4@studio.unibo.it (E.G.); davide.gori4@unibo.it (D.G.); 2 Regional Authority for Healthcare and Welfare, Emilia-Romagna Region, 40126 Bologna, Italy; dario.tedesco@regione.emilia-romagna.it; 3 Pituitary Unit, IRCCS Istituto delle Scienze Neurologiche di Bologna, 40139 Bologna, Italy; federica.guaraldi@yahoo.it

**Keywords:** coronavirus, COVID-19, global vaccination, healthcare workers, SARS-CoV-2, vaccination attitude, vaccine acceptance rate, vaccine hesitancy, vaccine intention, vaccine rejection

## Abstract

Vaccination currently appears to be the only strategy to contain the spread of COVID-19. At the same time, vaccine hesitancy (VH) could limit its efficacy and has, therefore, attracted the attention of Public Health Systems. This systematic review aimed at assessing anti-COVID-19 vaccine acceptance rates worldwide and at identifying populations more prone to vaccine hesitancy, for which specific interventions should be planned. PubMed database was searched using a purposely formulated string. One hundred out of the 9243 studies retrieved were considered pertinent and thus included in the analyses. VH rate was analyzed according to patient geographical origin, ethnicity, age, study setting, and method used for data collection; data from specific populations were separately analyzed. Overall, this study demonstrated significant differences in terms of VH in the general population and in the specific subgroups examined according to geographical, demographic factors, as well as associated comorbidities, underlining the need for purposely designed studies in specific populations from the different countries, to design targeted programs aimed at increasing awareness for confidence and complacency toward COVID-19 vaccines.

## 1. Introduction

Vaccine hesitancy (VH), defined by the SAGE working group as “delay in acceptance or refusal of vaccination despite availability of vaccination services” [1], has been recognized as a major threat to the effectiveness of this public health strategy aimed at containing and eradicating infectious diseases, deserving inclusion among major health concerns by the World Health Organization (WHO) in 2019 [2]. 

The potential consequences of VH are currently considered even more alarming because of the ongoing COVID-19 pandemic, against which vaccination appears to be the most efficacious strategy. Indeed, in addition to the considerable logistic and financial challenges implied in the organization of mass vaccination campaigns, VH could significantly limit or delay vaccination spread, thus preventing the rapid achievement of immunization rates required in the population (estimated at around 70% for COVID-19) [3] to fight the pandemic. 

Many studies have investigated the distribution and determinants of VH, and highlighted geographical differences, with high income European countries struggling with overall high rates [4]. At the same time, other groups at a higher risk for VH have been identified, including countries with low income and education [5,6].

In this context, this study aims to systematically review literature on vaccine acceptance, to define more precisely the extent of VH associated with the ongoing vaccination campaign against COVID-19, and thus try to identify subpopulations at increased risk for VH, deserving target strategies.

## 2. Materials and Methods

The study was conducted following the Preferred Reporting Items for Systematic Reviews and Meta-Analyses (PRISMA) [7]. Article search was performed using the search string: (COVID-19 OR COVID 19 OR COVID* OR Coronavirus* OR coronavirus* OR SARS-CoV-2) AND (vaccin* accept* OR vaccin* hesitanc* OR vaccin* resistance OR intent* vaccine OR vaccin* confidence OR hesitanc* OR vaccin* rejection OR vaccin*) on PubMed database. Asterisks were used to include any desinence of the most relevant words in our search. 

Original articles written in English, performed in adults of both genders, containing sufficient data of interest, and published from November 2019 to March 2021 were considered. Reviews, metanalysis, commentaries, editorials, and original studies not reporting the rate of vaccine acceptance/hesitancy toward COVID-19 vaccination were not included. Information on publication date, time period of data collection date, study design and setting, country, sample, gender and ethnicity of respondents and acceptance/hesitancy rate were collected from studies of interest. “Acceptance rate” was calculated considering both positive and uncertain answers showing willingness to be vaccinated (e.g., “probably yes”, “somewhat likely”, or “somewhat agree”). 

Geographical distribution was defined according to the 6 regions identified by the WHO (i.e., Africa, Americas, South-East Asia, Europe, Eastern Mediterranean, and Western Pacific) [8]. After customized analyses, worldwide distribution of VH/vaccine acceptance rate was obtained. Age was collected as mean, otherwise as interval. According to the setting, studies were divided between studies performed in a hospital, and/or in an extra-hospital context. Finally, the method used to collect patient data (i.e., during patient access to healthcare services, online, telephone or email) was recorded. Data were collected and analyzed using Microsoft Excel (Microsoft Corporation, Redmond, WA, USA).

## 3. Results

**Figure 1:**
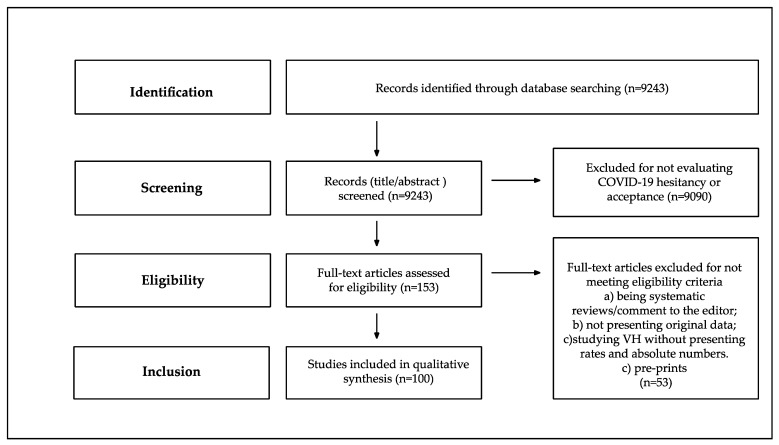
Flow chart of the study selection process.

One hundred of the 9243 identified records were considered of interest and, thus, included in the analyses (Figure 1). All studies were performed in 2020, except for five for which the period of data collection was not reported. Among the studies included in our review, 86 (86%) were cross-sectional, and 8 (8%) longitudinal, while the study type was not reported for 6 (6%) of the studies.

A sensitivity analysis was performed in a representative sample of the studies selected in order to test heterogeneity in the groups using I^2^ Statistics. Due to the high heterogeneity that emerged (>50%), the meta-analyses was not conducted.

A time-lead stratification analysis was not conducted since samples between pre- and post- approval of vaccination were not equally distributed. The vast majority of the studies 47/49 (96%) were, in fact, conducted in 2020 before approval of the vaccine and beginning of the vaccination programs.

### 3.1. Study Sample Characteristics

Main sample features of the studies included in the analysis are shown in Table 1 [9,10,11,12,13,14,15,16,17,18,19,20,21,22,23,24,25,26,27,28,29,30,31,32,33,34,35,36,37,38,39,40,41,42,43,44,45,46,47,48,49,50,51,52,53,54,55,56,57,58,59,60,61,62,63,64,65,66,67,68,69,70,71,72,73,74,75,76,77,78,79,80,81,82,83,84,85,86,87,88,89,90,91,92,93,94,95,96,97,98,99,100,101,102,103,104,105,106,107,108]. Patient numerosity varied widely among the studies (87–28,629 [32,45]).

Regarding gender distribution, female predominance was observed in 87 studies (n = 87) [10], and male predominance in 12, being the highest male prevalence (91.3%) observed in the study by Caban-Martinez et al. performed in US firefighters and health care workers (HCWs) [29]. Males and females were equally distributed in the study by Guidry et al. [10]. 

Mean sample age was reported as interval in 25 (25%) studies, and as mean in 38 (38%) studies, while this information was not given in 37 (37%). Ethnicity was reported in 43 (43%) studies.

### 3.2. Anti-COVID19 Vaccine Acceptance Rate in the General Population and in Specific Target Populations

Forty-nine (49%) studies were performed in the general adult population; 22 (22%) included HCWs, 9 (9%) patients with chronic diseases (i.e., HIV, multiple sclerosis, chronic respiratory diseases, kidney insufficiency requiring dialytic treatment, neoplasia, rheumatological diseases, and conditions induced by substance use) and their caregivers, 8 (8%) university students, 4 (4%) parents/guardians, 2 (2%) factory workers, 1 (1%) pregnant women and 1 (1%) SARS-CoV-2 positive patient. Five studies (5%) did not target any specific population. Results regarding the vaccine confidence rate for each study are shown in Table 1.

#### 3.2.1. General Population

According to the analysis performed in the general population, the lowest rates of vaccine confidence were found in Hong Kong (4.2–38%), Middle East Area (Jordan and Kuwait, 29.4% and 36.8%, respectively [79,80]) followed by the Democratic Republic of Congo (15.4%) [76,90]. On the other hand, the highest acceptance rate (94.3%) was reported in Malaysia [99]. 

#### 3.2.2. Specific Populations

In the group of HCWs, the lowest confidence rate (27.7%) was reported in the Democratic Republic of Congo [77], followed by the US (36%) [30], while the highest rate (96.2%) was reported in Asia (China, India, Republic of Indonesia, Singapore, Vietnam and Bhutan) [102].

When considering patients affected by chronic diseases and caregivers, the lowest confidence rate was found among American adults affected by substance use diseases (45%) [32], while the highest (85.6%) was reported in elderly affected by chronic respiratory diseases [44]. 

Among university students, the lowest acceptance rate (34.9%) was found in Egypt [37], and the highest in the US (98%) [78].

Gender distribution was analyzed in 84 (84%) studies, while 2 (2%) studies analyzed vaccine acceptance in the female population and 14 (14%) studies did not report this data.

Finally, Skjefte et al. reported a confidence rate of 52% among pregnant women and of 75.8% among parents/guardians in Australia [104].

### 3.3. Differences among WHO Regional Areas: General Population and HCWs

Most of the studies were conducted in the Region of the Americas (n = 35, 35%; 33 in the US; one in the US and Canada, and one in Mexico), followed by the European Region (n = 31; 31%), the Western Pacific Region (n = 15; 15%), the Eastern Mediterranean Region (n = 7; 7%), the African Region (n = 3; 3%) and the Southeastern Asian Region (n = 2; 2%). Finally, seven studies included patients from more than one region (Table 1).

In the European Region, the confidence rate varied from 40.9% to 92.3% in the general population, and from 54.9% to 95.1% in HCWs [56,64,65,66].

In the Region of the Americas, vaccine confidence varied between 36% and 98% in the general population and HCWs [30,37]. 

For the African Region, it was only identified by a study by Dinga et al. showing a confidence rate of 15.4% in the general population [76], and another by Kabamba Nzaji et al. reporting an acceptance rate of 27.7% in HCWs [77]. 

In the Eastern Mediterranean Region, confidence rate in the general population varied from 29.4% to 64.7% [80,82]; no study focused on HCWs. 

In the Western Pacific Region, the various studies reported confidence rates from 4.2% to 94.3% in the general population [90,99]; a single study focusing on nurses showed a confidence rate of 40% [89]. 

According to the two studies available for the South Eastern Asian Region, confidence rates were 94.3% in HCWs and 93.3% for the general population [100,101].

Studies including more than one Region showed confidence rates of 71.6–96.2% for HCWs, and of 71.5–80% for the general population [102,103,106,107].

**Figure 2:**
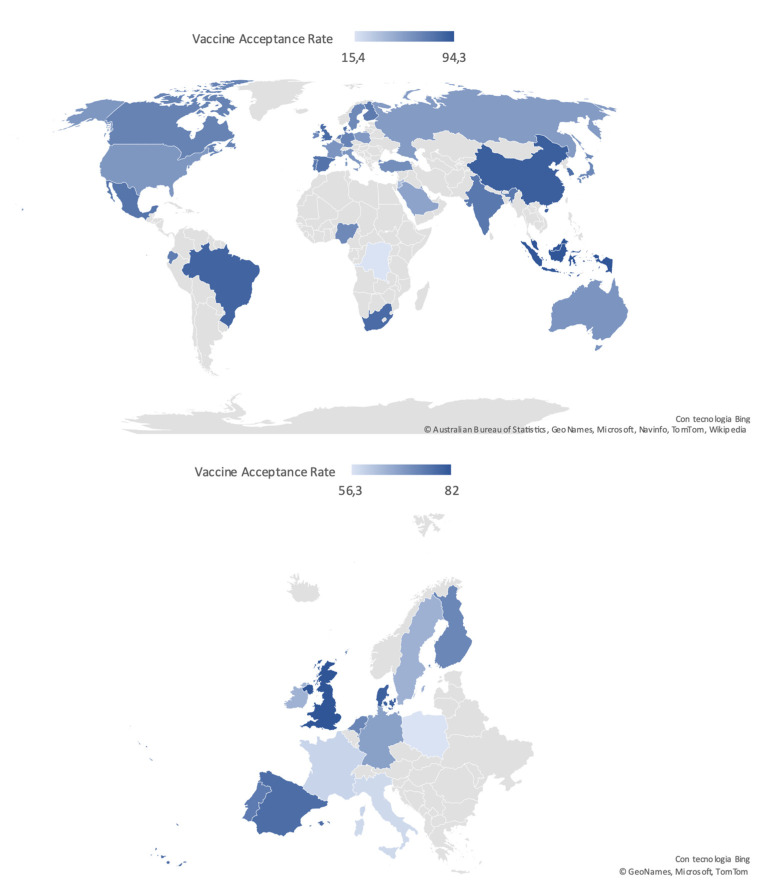
Worldwide and European distribution of COVID-19 vaccine acceptance rates in the general population, defined according to the most recent available data.

Acceptance rate distribution reported in the general population worldwide and in the European Countries is depicted in Figure 2.

## 4. Discussion

COVID-19 infection has represented a major health plague worldwide since 2020, for which vaccination currently appears to be the only efficacious strategy to achieve disease control. In this context, vaccine hesitancy (VH), responsible for outbreaks and epidemics of other infectious diseases (i.e., measles) secondary to the drop of immunized people under the herd immunity threshold [109], represents a major threat, deserving rapid identification of predisposing factors, and targeted countermeasures. This systematic review demonstrated largely variable rates of vaccine acceptance worldwide, in the general population, as well as in specific sub-populations. 

### 4.1. Gender Distribution

In most of the selected studies females were more represented than males. A higher prevalence of VH for the different vaccines has been widely reported in literature [110], and it has been hypothesized that this may be due to a lower trust in the Institutions and in the scientific community, an important driver of VH, in women [111]. With regards to COVID-19, the higher prevalence of severe infections and death among males [112] could have increased male risk perception and, subsequently, vaccine acceptance. At the same time, it has to be remarked that this study did not show a clear correlation between the number of males included in the studies surveys and vaccine acceptance. Therefore, several other factors might have influenced this relation.

### 4.2. Health Care Workers

Extremely variable vaccine confidence rates were reported among health care workers (HCWs). Following the geographical distribution, the lowest rates were observed in the Democratic Republic of Congo (27.7%) and the US (36.0%), and the highest (96.2%) in Eastern Asian countries (China, India, Republic of Indonesia, Singapore, Vietnam, and Bhutan) [30,77,102]. High rates of VH among HCWs are particularly alarming for their crucial role during the SARS-CoV-2 pandemic, in terms of daily patient care but also health literacy promotion for the general population. The wide range of confidence rates towards the COVID-19 vaccination found globally could be explained considering the different roles and relation to the patients played by the different HCWs (e.g., physician, nurse, pharmacist or clerical worker) [113]. Indeed, HCWs involved in direct patient care were found to be more confident, suggesting that an increased vaccine hesitancy could be related to a less direct contact with the patient and, consequently, a reduced risk perception of COVID-19 associated morbidity [34]. At the same time, the high rates of VH reported even in HCWs directly involved in patient care [30] underlines the need of interventions specifically addressed to this category of workers to increase their awareness towards the risk of unsuccessful vaccine campaigns [114].

### 4.3. Patients Affected by Specific Diseases 

Some studies showed a higher vaccine confidence rate in patients with chronic diseases as compared to the general population, as might be expected (i.e., 80.9% and 84.5% in patients with multiple sclerosis from Portugal and the US, respectively; or 85.6% in those with chronic respiratory diseases from UK), possibly secondary to the perception of frailty towards severe disease and death [115,116], which is true especially for respiratory diseases [117] and MS [118].

Other studies, focusing on oncologic (53.7%) and rheumatologic patients (54.9%), showed acceptance rates similar to the general population living in the same countries. Remarkably, some subpopulations showed particularly low acceptance rates, i.e., black adults with HIV (46%), people with drug addiction (45%), adults undergoing chronic dialysis (49%), and, finally, SARS-CoV-2 positives. Patients who had already been infected by the virus could possibly perceive themselves as immunized against COVID-19, independently from its variants, thus reducing the perceived advantages associated with vaccination and the willingness to be vaccinated. In the remaining categories, reluctance to accept a vaccine against COVID-19 might be due to the confounding effect of low income and education, which have been shown to be risk factors for chronic kidney disease, substance abuse, and HIV infection [119,120,121], or, finally, to the possible wariness of frail patients towards potential side effects of the vaccines.

### 4.4. Surveys Administration Setting

Almost all surveys were conducted online, sending questionnaires via e-mail or using other social media. The dissemination of these versatile information tools has undoubtedly facilitated and speeded up the data collection process, especially during COVID-19 pandemics, in the targeted populations. On the other hand, their use could be responsible for population selection bias, e.g., exclusion of the elderly, and problems in questionnaire filling/misunderstandings, due to the lack/difficulty of interaction with researchers.

Finally, online platforms (in particular the new social media) are considered by some recent studies as carriers of disbelief and skepticism about vaccines [122,123,124], potentially leading to higher rates of hesitancy in the average users of these online platforms. 

All these aspects could contribute to the variability of vaccine confidence rates. 

### 4.5. Geographical Distribution

The prevalence of vaccine acceptance/refusal widely varied across countries and WHO Regions, supporting the definition of VH given by the SAGE Group as “complex and context specific, varying across time, place and vaccines” [1]. This could be explained by the complex and unpredictable interaction of demographic, cultural, and social factors, including previously mentioned demographic and educational ones, but also people’s confidence in health and other governmental institutions, and, on the other hand, the tightness of governmental control on social and information media. 

The lowest acceptance rates among the general population were observed in Hong Kong, ranging from 4.2% to 38% depending on the type of vaccine evaluated in the study, and in the Democratic Republic of the Congo, corresponding to 15.4%. These two countries present vastly different cultures and demographic features, but share a recent history of political instability that should be considered while examining VH rate under a socio-political perspective.

Conversely, the highest rates were found in China (91.9%), Malaysia (94.3%), and Indonesia (93.3%). These results could reflect a higher awareness toward the terrible consequences of SARS-CoV-2 virus diffusion, as these Asian countries were among the first to be hit by the COVID-19 pandemic, and have consequent confidence in vaccines. 

The European Region was characterized by a very broad variability of vaccine acceptance rates, with the highest rates reported in United Kingdom (82%), and the lowest in Italy (40.9%) and in France (53.7%), reflecting once again the impact of cultural and sociodemographic heterogeneity of the examined geographical area on VH [12]. 

### 4.6. Limitations and Strengths

As previously mentioned, the main limitation of the study is represented by the great heterogeneity of the studies included, despite the selection performed. Moreover, some of them lack important methodological/patient information. For these reasons it was opted for a systematic review instead of a metanalysis. 

On the other hand, this work presents some strengths. First, the use of a very inclusive search string aiming to be as sensitive as possible. Thanks to this strategy the analysis started from a considerable pool of articles (more than 9000) reducing the risk of losing important data, that were then carefully analyzed to remove preprints or studies not presenting original data, thus increasing the study sensitivity while refining the research. Finally, data collected from studies of interest were analyzed according to some demographic, geographic, clinical, and social factors, to try to highlight major determinants of vaccine hesitancy. 

## 5. Conclusions

VH is a global and increasingly wide-spreading phenomenon. Nonetheless, remarkable differences in vaccine acceptance rates can be observed across countries and subpopulations, supporting the underlying complex and unpredictable interplay among demographic, geopolitical and cultural aspects, which are hard to be understood and discriminated. At the same time, focused research aimed at formulating targeted strategies to improve vaccine awareness, confidence and acceptance prevalence is of utmost importance.

Since communication, especially via social media, has been clearly demonstrated to play a pivotal role in determining adherence to vaccination, these instruments should be better exploited by the governmental institutions and the scientific community to increase people trust in the evidence-based rationale and rigorous production process, as well as expected short and long-term benefits of universal vaccination for the ongoing COVID-19 and potential future pandemics. 

## Figures and Tables

**Table 1:** COVID-19 confidence rates by included studies and sorted according to WHO global regional area definitions between November 2019 and March 2021.

WHO Region	Author	State	Data Collection	Study Design	Age (Mean or Most Represented Age Group)	Participants (n)	Males (%)	Ethnicity	Target Population	Setting	Confidence Rate
**Americas**											
	Fisher KA et al. [9]	US	16–20 April 2020	CS	48	991	48.5	Yes	Adults	Panelist	57.6
	Guidry JPD et al. [10]	US	July 2020	CS	46	788	50.0	Yes	Adults	Online	59.9
	Ruiz JB et al. [11]	US	15–16 June 2020	CS	n.a.	804	46.4	Yes	Adults	Panelist	62.2
	Callaghan T et al. [12]	US	28 May–8 June 2020	CS	45	5009	49.5	Yes	Adults	Survey Platform	67.7
	Meyer MN et al. [13]	US	December 2020	CS	43	16,292	n.a.	No	HCWs	E-mail	55.3
	Kreps S et al. [14]	US	July 2020	CS	43	1971	49.3	No	Adults	Online	56.0
	Bogart LM et al. [15]	US	May–July 2020	CS	50	101	87.0	Yes	Black americans; HIV+	Online	46.0
	Reiter PL et al. [16]	US	May 2020	CS	n.a.	2006	43.3	Yes	Adults	Online	69.0
	Khubchandani J et al. [17]	US	June 2020	CS	n.a.	1878	48.5	Yes	Adults	Online	79
	Meier BP et al. [18]	US	28–30 October 2020	CS	45	1072	47.9	No	Adults	Online	70.0
	Graupensperger S et al. [19]	US	2–13 November 2020	CS	20	647	35.2	Yes	Students	Online	91.6
	Salmon DA et al. [20]	US	n.a.	CS	n.a.	2525	48.2	Yes	Adults	Online	50.0
	Ehde DM et al. [21]	US	10 April–6 May 2020	CS	n.a.	486	17.3	Yes	Adults with Multiple Sclerosis	Online	84.5
	Pogue K et al. [22]	US	n.a.	CS	n.a.	316	50.3	Yes	Adults	Online	68.6
	Daly M et al. [23]	US	1 April–31 October 2020	LS	47	7547	47.9	Yes	Adults	Online	71.0 (April 2020); 53.6 (October 2020)
	Allen JD et al. [24]	US	13 April–8 June 2020	CS	27–45	396	0	Yes	Women aged 27–45	Online	56.8
	Latkin C et al. [25]	US	1st survey 24–27 March 2020, 2nd survey 5–14 May 2020, 3rd survey 22–23 July 2020	LS	n.a.	592	44.0	Yes	Adults	Online	59.1
	Malik AA et al. [26]	US	May 2020	CS	n.a.	672	43.0	Yes	Adults	Online	67.0
	Manning ML et al. [27]	US	10 August–14 September 2020	CS	n.a.	1212	28.0	Yes	Nurses; Nursing Students	Online	60.0 (Full-time faculty); 45.0 (Adjunct faculty and students)
	Schrading WA et al. [28]	US	4 January 2021	LS	30–39	1398	35.0	Yes	HCWs	Hospital	86.0
	Caban-Martinez AJ et al. [29]	US	1–31 October 2020	CS	40–49	3169	91.3	Yes	Firefighters; HCWs	Online	48.2
	Shekhar R et al. [30]	US	7 October–9 November 2020	CS	31–40	3479	24.8	Yes	HCWs	Online	36.0
	Rungkitwattanakul D et al. [31]	US	n.a.	n.a.	n.a.	90	60.0	Yes	Adults on dyalisis	Hospital	49.0
	Mellis AM et al. [32]	US	10–27 September 2020	n.a.	43	87	46.0	No	Adults with Substance Use Disorders	Telephone; Video-conferencing	45.0 (Immediate readiness); 8.0 (Readiness after a delay)
	Amin DP et al. [33]	US	11–23 January 2021	CS	41–50	240	43.0	Yes	HCWs	Online	92.0
	Kuter BJ et al. [34]	US	November–December 2020	CS	na.	12,034	21.0	Yes	HCWs	E-mail	63.7
	Unroe KT et al. [35]	US	14–17 November 2020	CS	41–60	8243	12.6	Yes	HCWs	Text message; E-mail	45.0
	Gatwood J et al. [36]	US	3–8 June 2020	CS	25–44	1000	49.0	Yes	Adults	Online	54.1
	Lucia VC et al. [37]	US	n.a.	CS	n.a.	168	43	No	Medical students	Online	98.0
	Pamplona G et al. [38]	US	13–21 January 2021	n.a.	n.a.	157	n.a.	No	HCWs	Hospital and Home-care	96.2
	Kociolek LK et al. [39]	US	21 December 2020–13 January 2021	CS	≤40	4448	17.0	Yes	HCWs	E-mail	59.8
	Gadoth A et al. [40]	US	24 September–16 October 2020	CS	30–49	540	28.3	Yes	HCWs	Hospital	33.2 (Immediately); 65.5 (After a delay)
	Shaw J et al. [41]	US	23 November–5 December	CS	42	5287	27.2	Yes	Adults	Hospital	57.5
	CastañedaasVasquez DE et al. [42]	Mexico	October–December 2020	CS	21 (median age)	543	32.8	Yes	HCWs	Online	94.5
	Taylor S et al. [43]	US; Canada	6–19 May 2020	CS	53	3674	57.0	Yes	Adults	Online	80.0 (Canadians); 75.0 (U.S.)
**Europe**											
	Williams L et al. [44]	UK	April 2020	CS	70 (older adults), 43 (participants with chronic respiratory patologies)	527	43.3	No	Elderly with chronic respiratory diseases	Online	85.6
	Jackson SE et al. [45]	UK	7 September–5 October 2020	CS	n.a.	28,629	n.a.	No	Adults	Online	65.8 (Non-smokers); 66.0 (Ex-smokers) e 50.9 (Smokers)
	Williams L et al. [46]	UK	1st survey: 20 May 12 June 2020, 2nd survey: August 2020	LS	n.a.	3436 (1); 2016 (2)	19.4 (1); 17.6 (2)	Yes	Adults	Online	74.0 (1); 78.0 (2)
	Sherman SM et al. [47]	UK	14–17 July 2020	CS	46	1,5	49.0	Yes	Adults	Online	64.0
	Freeman D et al. [48]	UK	24 September–17 October	CS	47	5114	50.3	Yes	n.a.	Online	71.7
	Batty GD et al. [49]	UK	4 November 2020	LS	53	11,955	n.a.	No	n.a.	Outpatient	82.8
	Robertson E et al. [50]	UK	24 November–1 December 2020	LS	55–64	12,035	38.8	Yes	Households	Online	82
	Gagneux-Brunon A et al. [51]	France	26 March–2 July 2020	CS	30–49	2047	26.0	No	HCWs	Social Media; E-mail; Hospital Website and Written Surveys	76.9
	Ward JK et al. [52]	France	April 2020	CS	35–64	5018	56.6	No	Adults	Panelist	76.0
	Detoc M et al. [53]	France	26 March–20 April 2020	CS	30–49	3656	32.6	No	Adults	Social Media; E-mail; Hospital Website and Written Surveys	77.6
	Montagni I et al. [54]	France	8–11 May 2020	CS	28	1644	21.5	No	Adults	Online	70.5
	Barrière J et al. [55]	France	11 November–12 December 2020	CS	67 (median)	536	44.0	No	Oncological patients	Outpatient	53.7
	Green MS et al. [56]	Israel	October 2020	CS	n.a.	957	45.0	Yes	Adults older than 30	Panelist	59.0–92.3
	Dror AA et al. [57]	Israel	March 2020	CS	n.a.	1941	n.a.	No	HCWs; Adults	Online	75.0
	Zigron A et al. [58]	Israel	March–April 2020	CS	n.a.	506	43.0	No	Dentists	Online	85.0
	La Vecchia C et al. [59]	Italy	16–28 September 2020	CS	≥55	1055	48.2	No	Adults	Panelist	53.7
	Prati G et al. [60]	Italy	April 2020	CS	32	624	46.0	Yes	Adults	E-mail; Online; Social Media	75.8
	Barello S et al. [61]	Italy	n.a.	CS	23	934	20.4	No	University students	E-mail	86.1
	Di Gennaro F et al. [62]	Italy	October 2020	CS	36	1723	47.0	No	HCWs	Online	67.0
	Graffigna G et al. [63]	Italy	n.a.	CS	44	1004	49.0	No	Adults	Online	58.6
	Gerussi V et al. [64]	Italy	September–November 2020	CS	53	599	46.6	No	Adults SARS-CoV-2 positive	Telephone	40.9
	Pastorino R et al. [65]	Italy	8 June–2 July 2020	CS	n.a.	436	n.a.	No	University students	Online	95.1
	Priori R et al. [66]	Italy	n.a.	CS	n.a.	626 (Rheumatological Patient); 345 (Control Group)	n.a.	No	Adults with rheumatological diseases	Online	54.9
	Barello S et al. [67]	Italy	27 November–3 December 2020	n.a.	n.a.	1005	n.a.	No	Adults	Online	58.0
	Karlsson LC et al. [68]	Finland	1st survey: May 2020–June 2020, 2nd survey: 30 March 2020–14 April 2020 3rd survey: 3–17 April 2020	CS	n.a.	825 (S1); 205 (S2); 1325 (S3)	21.2 (S1); 30.7 (S2); 19.0 (S3)	Yes	Parents/Guardiands	Online	73.9 (S1); 77.8 (S2); 72.9 (S3)
	Szmyd B et al. [69]	Poland	22–25 December 2020	CS	20 (median)	1971	12.3 (Medical students); 36.9 (Non-medical students)	Yes	University students	Online	92.0 (Medical students); 59.4 (Non-medical students)
	Feleszko W et al. [70]	Poland	2–9 June 2020	CS	n.a.	1066	n.a.	No	Adults	Online	72.0
	Papagiannis D et al. [71]	Greece	15–22 December 2020	CS	48	340	51.2	No	HCWs	Hospital	78.5
	Serrazina F et al. [72]	Portugal	21 December 2020–3 January 2021	CS	45	256	27.0	No	Adults with Multiple Sclerosis	Hospital; Online	80.9
	Eguia H et al. [73]	Spain	10 September–23 November 2020	CS	51	731	44.0	No	Adults	Social Media	77.6
	Yigit M et al. [74]	Turkey	n.a.	CS	40	428	36.4	No	Parents/Guardians	Social Media; In-person	62.6 (National vaccine); 33.9 (International)
**African Region**											
	Ditekemena JD et al. [75]	DRC	24 August–8 September 2020	CS	35	4131	31.6	No	n.a.	Online	55.9
	Dinga JN et al. [76]	DRC	5 August 2021	CS	n.a.	2512	45.1	No	Adults	Online; In-person	15.4
	Kabamba Nzaji M et al. [77]	DRC	March–April 2020	CS	40	613	50.9	No	HCWs	Hospital; Online	27.7
**Eastern Mediterranean Region**											
	Saied SM et al. [78]	Egypt	08–15 January 2021	CS	20	2133	34.8		Medical students	Online	34.9
	Al-Qerem WA et al. [79]	Jordan	October 2020	CS	18–29	1141	33.5	No	Adults	Social Media	36.8
	Sallam M et al. [80]	Jordan; Kuwait	14–18 December 2020	CS	24 (Jordan), 30 (Kuwait)	Giordania, 2173; Kuwait 771; Saudi Arabia 154	30.6 (Jordan); 36.1 (Kuwait); 23.4 (Saudi Arabia)	Yes	Adults	Online	29.4
	Qattan AMN et al. [81]	Saudi Arabia	08–14 December 2020	CS	n.a.	673	n.a.	No	Adults	Online	50.5
	Al-Mohaithef M et al. [82]	Saudi Arabia	n.a.	CS	n.a.	992	34.2	No	Adults	Online	64.7
	Alqudeimat Y et al. [83]	Kuwait	26 August–1 September 2020	CS	n.a.	2368	31.8	No	Adults	Online	53.1
	Alabdulla M et al. [84]	Quatar	15 October–15 November 2020	CS	36–45	7821	59.43	No	n.a.	Online	60.5
**Western Pacific Region**											
	Dodd RH et al. [85]	Australia	17–21 April	CS	56–90	4362	n.a.	No	Adults	Online	85.8
	Edwards B et al. [86]	Australia	August 2020	LS	n.a.	3061	n.a.	No	Adults	Online, Telephone	58.5
	Faasse K et al. [87]	Australia	2–9 March 2020	CS	30–49	2174	23.1	Yes	Adults	Online; Social Media	81.1
	Rhodes A et al. [88]	Australia	15–23 June 2020	n.a.	n.a.	2018	n.a.	No	Parents/Guardians	Hospital; Online	75.8
	Wang K et al. [89]	Hong Kong SAR	February–March 2020	CS	30–39	806	19.3	No	Nurses	E-mail	40.0
	Yu Y et al. [90]	Hong Kong SAR	16–30 September 2020	CS	n.a.	450	31.0	No	Adults	Telephone	4.2–38
	Gan L et al. [91]	China	October–November 2020	CS	18–29	1009	37.9	No	Adults	Online	60.4
	Lin Y et al. [92]	China	May 2020	CS	26–35	3541	48.1	No	Adults	Social Media	83.3
	Wang J et al. [93]	China	March 2020	CS	n.a.	2058	45.8	No	Adults	Online	91.3
	Wang J et al. [94]	China	1st survey March 2020, 2nd survey November–December 2020	LS	n.a.	791	46.9	No	Adults	Online	91.9 (1); 88.6 (2)
	Zhang KC et al. [95]	China	1–7 September2020	CS	n.a.	2053	42.6	No	Factory workers	Online	80.6 (If 80.0% vaccine efficacy and free of charge)
	Mo PK et al. [96]	China	01–28 November 2020	CS	19	6922	36.4	Yes	University students	Online	78.9 (Vaccine for free); 60.2 (Vaccine fee-based)
	Zhang KC et al. [97]	China	1–7 September 2020	CS	n.a.	2053	37.5	No	Factory workers; Parents/Guardians	Online	72.6
	Yoda T et al. [98]	Japan	September 2020	n.a.	45	1100	53.0	No	Adults	Panelist	65.7
	Wong LP et al. [99]	Malaysia	3–12 April 2020	CS	31–40	1159	44.0	Yes	Adults	Social Media	94.3
**South-East Asia Region**											
	Parajuli J et al. [100]	Nepal	April–May 2020	CS	20–29	230	50.4	No	HCWs	Hospital	94.3
	Harapan H et al. [101]	Indonesia	25 March–6 April 2020	CS	n.a.	1359	34.3	No	Adults	Online	93.3
**More than one Region**											
	Chew NWS et al. [102]	China; India; Indonesia; Singapore; Vietnam and Bhutan	12–31 December 2020	CS	33	1,72	39.0	No	HCWs	Online; Telephone	96.2
	Lazarus JV et al. [103]	China; Brazil; South Africa; South Korea; Mexico; US; India; Spain; Ecuador; UK; Italy; Canada; Germany; Singapore; Sweden; Nigeria; France; Poland; Russia	16–20 June 2020	CS	25–54	13,426	45.8	No	Adults	Online; Telephone	71.5 Overall; China 88.6; Brazil 85.4; South Africa 81.6; South Korea 79.8; Mexico 76.3; US 75.4; India 74.5; Spain 74.3; Ecuador 71.9; UK 71.5; Italy 70.8; Canada 68.7; Germany 67.9; Singapore 67.9; Sweden 65.2; Nigeria 65.2; France 58.9; Poland 56.3; Russia 54.9
	Skjefte M et al. [104]	Worldwide	28 October–18 November 2020	CS	34	17,871	0	No	Pregnant women; Mothers	Online	52.0 (Pregnant women); 73.4 (Non-pregnant women)
	Goldman RD et al. [105]	US; Canada; Japan; Spain; Switzerland	26 March–31 May 2020	CS	40	1552	25.5	No	Caregivers	Online	65.2
	Neumann-Böhme S et al. [106]	Denmark; UK; Italy; Portugal; Netherland; Germany; France	April 2020	CS	n.a.	1000	n.a.	No	Adults	Online	80.0 Denmark; 79.0 UK; 77.3 Italy; 75.0 Portugal; 73.0 Netherlands; 70.0 Germany; 62.0 France.
	Verger P et al. [107]	France; Belgium; Canada	October–November 2020	CS	40–59	2678	30.8	No	HCWs	Online; Telephone; E-mail	71.6 Overall; France 46.5 (Certainly), 28.8 (Probably); Belgium 39.5 (Certainly), 36.5 (Probably); Canada 42.9 (Certainly), 27.6 (Probably)
	Murphy J et al. [108]	Ireland; UK	n.a.	CS	n.a.	1041 (Ireland); 2025 (UK)	48.2 (Ireland); 48.3 (UK)	Yes	Adults	n.a.	Ireland 65.0; UK 69.0

US: United States of America; UK: United Kingdom; DRC: Democratic Republic of Congo; CS: Cross Sectional study; LS: Longitudinal Study; HCWs: Health Care Workers.
